# Expressiveness and Instrumentality of Crime Scene Behavior in Spanish Homicides

**DOI:** 10.3390/ijerph16224526

**Published:** 2019-11-15

**Authors:** María del Mar Pecino-Latorre, María del Carmen Pérez-Fuentes, Rosa María Patró-Hernández, Jorge Santos-Hermoso

**Affiliations:** 1 Department of Psychology, University of Almería, 04120 Almería, Spain; marpecino@gmail.com; 2 Department of Psychology, Faculty of Psychology, Universidad Politécnica y Artística del Paraguay, 1628 Asunción, Paraguay; 3 Department of Psychology, University of Murcia, 30100 Murcia, Spain; rosapatro@um.es; 4 Institute of Forensic Sciences and Security, Autonomous University of Madrid, 28049 Madrid, Spain; jorge.santos@icfs-uam.es

**Keywords:** homicide, instrumental homicide, expressive homicide, delinquent, criminal profiling, crime scene behavior

## Abstract

One of the current trends in the study of criminal profiling consists of developing theoretical and methodological typologies to offer information of operational use in police investigations. The objective of this work was to verify the validity of the instrumental/expressive model, so as to establish homicide typologies based on modus operandi relationships, characteristics of the victims, and characteristics of perpetrators. The sample consisted of 448 homicide cases registered in the database of the Homicide Revision Project of the Office of Coordination and Studies of the Spanish Secretary of State and Security. Through multidimensional scaling and cluster analysis, three expressive homicide subtypes were identified (expressive-impulsive, expressive-distancing, and expressive-family), as well as two instrumental homicide subtypes (instrumental-opportunist and instrumental-gratification). The expressive homicide typologies accounted for almost 95% of all of the studied cases, and most of the homicides occurring in Spain were found to take place between individuals who know one another (friends, family members, intimate couples/ex-couples). The findings from this study suggest that the instrumental/expressive model may be a useful framework for understanding the psychological processes underlying homicides, based on the study of relationships between the crime and aggressor characteristics, which may be very helpful in the prioritization of suspects.

## 1. Introduction

The conventional police techniques used during criminal investigations contribute greatly to crime solving. The crime scene location is the best source of information, since sufficient information and evidence tends to be found here, so as to suitably reconstruct the crime events and gather information as to the identity of the potential perpetrator/s [1,2]. However, when insufficient forensic evidence is available, in the case of mortal victims who cannot provide a physical description of the offender/s, or when the relationship between the victim and the perpetrator is unknown, police investigations may be slowed down or even come to a complete halt in very complex cases [3,4].

Thus, criminal profiling has developed as a supplemental technique to assist criminal investigations, forming hypotheses regarding the potential characteristics of a crime’s perpetrator and helping to establish a more rigorous suspect prioritization and, therefore, increase the efficiency of police investigations [5,6,7].

Since the onset of criminal profiling, numerous empirical studies have been carried out on homicides (both serial and simple), since they are considered to be the most serious type of violent behavior [8,9,10]. Today, most research attempts to verify the basic assumptions of criminal profiling, that is, the hypothesis of consistency and homology as necessary and sufficient conditions to prove its validity and utility [11]. The consistency hypothesis refers to the similarity between the delinquent’s behavior when committing a crime and his/her every day, normal behavior [12]. So, for example, the works of Trojan and Salfati [13] and Youngs, Ioannou, and Eagles [14] have followed this line of study.

The homology hypothesis, on the other hand, sustains that the similarity between criminal behaviors will be determined by the similarity of delinquent characteristics [15]. Specifically, studies have focused on determining the characteristics that relate homicides with the aggressor without inferring psychological characteristics or constructs [16,17,18].

In parallel, throughout the history of criminal profiling, diverse homicide typologies have been created in order to ensure more rigorous and systematic criminal profile-generation processes [19,20,21,22]. Today, developing typologies that have an extensive theoretical foundation and that are based on intense methodology are considered essential, so as to support decision-making carried out during the criminal investigations [1,5,23,24].

Within this context, the instrumental/expressive model was developed as an attempt to overcome methodological and theoretical issues, and the absence of empirical validation for prior classification systems. This model was designed in order to create a classification system to help explain violent behavior, describing the interpersonal dynamics existing between victim–perpetrator, and to help to thematically differentiate between actions carried out by aggressors during homicides [25]. The idea of conceptualizing the homicide act based on the instrumental/expressive dichotomy has been supported by the works of Feshbach [26], who established two types of aggression according to the objectives and compensation derived from the homicide.

On the one hand, instrumental aggression is motivated by the desire to obtain some type of benefit (economic, sexual, power) and, although the aggressor’s main objective is not to cause physical harm to the victim, violence may be used whenever it is needed to achieve the desired results [26,27,28]. This type of aggression is related to homicides carried out during the course of other criminal activities (robbery, sexual assault).Victims may be most likely to be strangers in robberies, but not in sexual assault cases, who are perceived by the aggressor as a means to satisfying their psychological needs [29,30,31]; they are also characterized by higher levels of premeditation, emotional coldness, and control [32,33,34]. In addition, this type may be an indicator of pathology, since aggression is used as a means to achieve a specific purpose [27,28].

Expressive aggression, on the other hand, takes place in response to some sort of threat that is perceived by the aggressor, which may or may not be real. It is related to an intense emotional activation, anger and hostility, and the intent is to cause physical harm (or even death) to the victim [26,27,28,35]. When expressive aggression results in homicide, they tend to take place between individuals who know one another, often resulting from an escalation of violence, in which aggressive behavior is of an emotional nature, suggesting very impulsive and violent actions [36,37,38].

Prior studies have demonstrated that the majority of homicides may be classified in a dominant typology, with expressive homicides predominating over instrumental ones [32,34,36,39,40,41]. In fact, expressive aggression has been considered the most basic form of violent behavior, whereas instrumental aggression only tends to occur in a reduced number of aggressors [27,28].

Numerous studies have demonstrated the utility and effectiveness of this model for establishing a homicide typology and for linking these typologies with aggressor characteristics using a multivariate methodology on samples from distinct countries and cultures. Some of the most relevant works have been conducted in Belgium [31,32], Korea [33,42], the United States [13,30,34,43,44,45], Finland [36,37], the United Kingdom [14,27,28,40], and Serbia [29].

As for the methodology associated with the instrumental/expressive model, multidimensional scaling is the most frequently used statistical technique to identify behavior topics in homicide scenes [38,39,40,44,46], although correspondence analyses have also been found to be useful [30], as well as logistic regression analysis [47], and even a combination of different statistical procedures, such as multiple correspondence analysis, cluster analysis, and factorial analysis [29]. So clearly, as the volume of works has increased, the complexity of this classification has also grown, including new subtypes and the use of different statistical procedures to improve the classification of homicides and to offer more objective, replicable results [25].

Based on the literature review conducted on the instrumental/expressive model, no previous studies conducted in Spain have used this theoretical framework or similar statistical procedures to classify homicides based on typologies and to link them to aggressor characteristics. All of the relevant studies found in this area were conducted in other countries. Therefore, the objective of this work is to verify the validity of this instrumental/expressive classification model to thematically differentiate between homicide structure in Spain, and to establish typologies based on the relationships between the modus operandi, characteristics of the victims, and characteristics of aggressors.

## 2. Materials and Methods

### 2.1. Participants

The initial sample consisted of 684 homicides registered in Spain between 2010 and 2012 (both inclusive), of which, multiple homicide cases were discarded (those with more than one perpetrator and/or victim) (*n* = −213), those carried out by minors (*n* = −13), those that were not solved by the police (*n* = −1), and those in which no information was available (*n* = −9). Finally, the study sample included a total of 448 homicides, according to the typology established by the United Nations Office on Drugs and Crime [9], 89% (*n* = 388) were interpersonal homicides (including intimate partner or family relationships and other interpersonal relationships, in which the victim and perpetrator may or may not have known each other) and 11% (*n* = 48) were linked to other criminal activities “that are aimed, directly or indirectly, at obtaining illicit profits” (e.g., homicide committed in the perpetration of a robbery) [9] (p. 39). Of the entire sample, 90.8% (*n* = 407) of the perpetrators were males and 9.2% (*n* = 41) were females, having a mean age of 41.35 (SD = 15.24) and 39.24 (DT = 14.63), respectively.

### 2.2. Procedure

The data used in this study came from the Homicide Revision Project (HRP) [48]. This project was developed and coordinated by the Office of Coordination and Studies of the Spanish Secretary of State and Security of the Spanish Ministry for Home Affairs, in collaboration with the country’s armed forces (Civil Guard and National Police forces) and several Spanish universities.

During the early stages of the project, the project leaders requested reports from the corresponding police departments and created a database to permit information collection. Then, specialized training was received on how to carry out the data dump procedure and information extraction, ensuring the confidentiality and ethical treatment of the data (e.g., anonymized database). 

The final HRP database includes information on completed homicides that were known to national security forces during the period of 2010–2012, and it contains basic characteristics of each homicide, on the sociodemographic background of both victim and perpetrator, and on the perpetrator crime scene behavior.

Next, upon receiving authorization from the relevant official of the Office of Coordination and Studies of the Spanish Secretary of State and Security, the database was provided for the purposes of the present study. 

Later, the sample was selected, in accordance with the methodology used in studies on this area. Selected cases were simple homicides (those with one perpetrator and one mortal victim) [27,38], carried out by perpetrators over the age of 18 [29,32], and in which the police had managed to solve the crime [37,42]. 

Afterwards, data purging was carried out in order to thoroughly analyze the data quality and to prepare a matrix for statistical analyses, with the final sample consisting of 448 homicide cases. This was seen as a considerable sample size, given the data that tends to be available from police investigations.

Finally, variables were selected in accordance with prior studies that had used the instrumental/expressive model (Table 1). To prepare the data for the subsequent statistical analysis, categorical variables were transformed into dichotomous variables based on the presence (1) or absence (0) of these said behaviors or characteristics in the homicide.

### 2.3. Data Analysis

The first step of the analytical strategy was to use nonmetric multidimensional scaling (nMDS) with the R statistics software (package ‘smacof’) [49] to test the hypothesized two thematic structures in terms of instrumentality and expressivity, 49 variables reflecting the characteristics of the homicide, the modus operandi of the offender, and the characteristics of the offender and victim (Table 1). The nMDS is an exploratory data analysis technique that represents the correlations between variables as distances in a bidimensional map, it is based on the supposition that the underlying structure of the homicide will be more easily appreciated if examining the relationships between all of the variables simultaneously [14,37,50]. Thus, this procedure provides an overall view of the relationships between all of variables, where the proximity of these indicates the frequency of joint appearance and thereby, similarity [51], permitting analysis and interpretation of the psychological processes underlying the homicide [5,51,52].

The nMDS was carried out on an association matrix of Jaccard index [31,53]. It is considered to be the most suitable measure to treat data from police sources, which, having been collected for purposes other than research, tend to fail to include certain variables due to a lack of (police) interest, even when said variables were in fact present in a specific case [27,32,36]. Next, the model’s goodness of fit was assessed, examining the Kruskal stress I index, which ranges between 0 (perfect fit) and 1, and the Shepard diagram [54,55].

The second step was to use a multivariate statistical technique of *k*-medoids cluster analysis, as a complement to the nMDS, based on the R statistical software (package ‘cluster’). The purpose of this technique was to establishe distinct homicide typologies [56,57]. Thus, based on the coordinates matrix resulting from the nMDS, the variables were classified in *k* clusters based on the Manhattan distance [58]. To identify distinct homicide typologies, the number of clusters to be formed was established a priori, examining the optimum number of groupings that adjust to the theoretical foundations of the instrumental/expressive model. Next, the internal quality of the groupings was assessed, considering indicators such as homogeneity and separation, and specifically analyzing the overall silhouette and the Dunn index [58,59].

The third step was to determine the suitability of the instrumental/expressive classification model, whereby each of the analyzed homicide cases was assigned to a dominant cluster or topic. To do so, a proportional method was used based on the R statistical software (‘max.col function’), such that each case was assigned to the cluster having the greatest proportion of variables [31,33,60].

Finally, the results were interpreted based on the theoretical foundations of the instrumental/expressive model [5,28].

## 3. Results

### 3.1. Multidimensional Scaling

**Figure 1:**
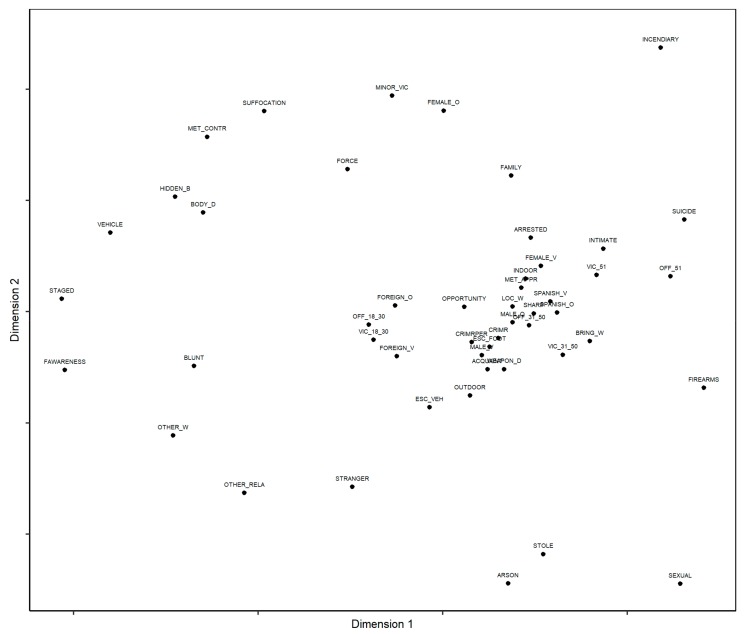
Bidimensional map of the nonmetric multidimensional scaling (nMDS).

Figure 1 shows the resulting bidimensional nMDS map, with each point corresponding to one of the 49 variables describing the characteristics of the homicide, the modus operandi of the offender, and the characteristics of the offender and victim as defined in Table 1. The spatial configuration of the variables appears, in which the proximity of these indicates the frequency of joint appearance, and, therefore, their thematic similarity. 

A *Stress*-I index of 0.228 was obtained, suggesting a poor data fit; however, in the Shepard diagram, the points fit well to the regression line, leading to an increasing monotone function [54]. Given that the stress value is not a conclusive criterion for determining the fit of the data, it may be assumed that the model has an acceptable goodness of fit. In fact, some authors have suggested that it is possible to accept a MDS model that does not have perfect fit, assuming that the representation of the variables permits a significant interpretation of the data [38,39].

### 3.2. Cluster Analysis

Before proceeding with the cluster analysis to establish the homicide typologies, five optimal groupings were established, since this adjusted to the theoretical foundations of the instrumental/expressive model and notably improved the variable grouping. Similarly, based on the analyses carried out to assess the internal quality of the groupings, it may be assumed that the clusters have an acceptable internal validity, ensuring the fit of the simple homicide typologies that were established.

**Figure 2:**
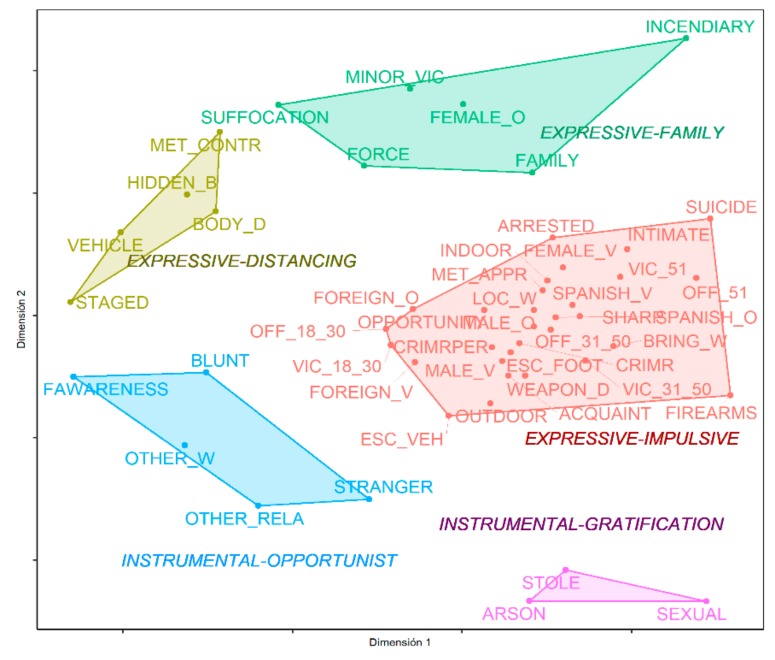
Simple homicide typology according to the instrumental/expressive model.

As Figure 2 shows, there were five subregions that could be distinguished on the plot: expressive-distancing, expressive-family, expressive-impulsive, instrumental-opportunist, and instrumental-gratification.

#### 3.2.1. Expressive-Impulsive Homicides

This typology is considered to be the most basic form of violent behavior and therefore, it is one of the most predominant characteristics of the 448 homicides. Specifically, most of these homicides take place in interior locations (65.2%), using opportune weapons (53.4%), often with sharp weapons (50.6%) that tend to be located by police during the investigation (85.6%). Aggressors tend to be male (90.8%), having Spanish nationality (70%), aged between 31 and 50 (50.6%) and with a past criminal record (59.1%). Victims tend to be male (53.2%) and of Spanish nationality (72%).

These homicides are characterized by a lack of planning and premeditation, and violent and impulsive behavior of an emotional nature (e.g., opportune weapons, sharp weapons, offender detained at the crime scene). The victim’s body tends to be found on the site of the act, revealing a lack of intent by the perpetrator to hide or destroy the evidence of the crime. Victims tend to be individuals who are known by the perpetrator, making it likely that these are precipitated homicides caused by an argument or conflict with the victim, with the aggressor attempting to inflict pain, thus suggesting the important role of emotions in these cases.

Likewise, three subtypes of expressive-impulsive homicides are found. The first refers to cases in which both the victim as well as the perpetrator are aged between 18 and 30 years, and both are non-Spanish nationals.

The second type refers to perpetrators with criminal records against people, demonstrating a criminal behavior that is coherent with his/her criminal record. In these cases, the offender tends to bring a weapon to the crime scene, often an armed weapon, which is disposed of after committing the crime. These homicides tend to take place in outdoor locations, where the perpetrator flees from the crime scene by foot or in a vehicle. As for the victims, they tend to be between the ages of 31 and 50.

The third case refers to homicides taking place between couples. The victims tend to be females, over the age of 51, who have an intimate relationship with the aggressor (past or present). Aggressors tend to be in the same age range as the victims, are usually detained at the scene, and/or commit suicide or attempt to do so following the crime.

#### 3.2.2. Expressive-Distancing Homicides

The second typology is made up of a subtype of expressive homicides in which the presence of postmortem actions related to the hiding and displacement of the victim’s body from the original crime scene are noteworthy. These acts reflect the perpetrator’s intent to distance him/herself from the crime scene and to disassociate him/herself from the criminal act, as well as a need to dispose of any physical evidence existing at the crime scene (e.g., a staged scene). The homicides tend to be carried out inside vehicles; furthermore, the use of methods to control the victim suggests that the offender needs to use additional mechanisms in order to achieve her submission (e.g., gagging, tying up, presence of a weapon) to successfully carry out the crime, most likely due to his/her close relationship to the victim.

#### 3.2.3. Expressive-Family Homicides

The third typology consists of a subtype of expressive homicides that are carried out in the family setting, mainly in the form of filicides, in which females tend to be the perpetrators and the victims tend to be minors. This type of homicide has certain precipitated, emotional characteristics, resulting from emotional discharges that may be due to psychological and external stressors (e.g., negative moods, annoyance, fury), in which the perpetrator attempts to satisfy his/her emotional needs. Thus, suffocation and the use of physical force are the main methods used to carry out the homicide, being indicative of the emotional relationship between the victim and the aggressor.

#### 3.2.4. Instrumental-Opportunist Homicides

The fourth typology consists of an instrumental subtype, in which the violent act carried out by the aggressor is precipitated, based on an ulterior motive (sensation of power, control). The victims tend to be strangers and/or have other types of relationships with the aggressors (e.g., prostitutes), and are perceived as objects for which the aggressor has no sort of feeling. Therefore, it is likely that they are opportune victims, having a certain vulnerability to being victimized. Blunt objects and other types of weapons are the most common causes of death; furthermore, the aggressor is characterized by a forensic knowledge, not leaving physical evidence or biological remains at the crime scene.

#### 3.2.5. Instrumental-Gratification Homicides

The fifth and final typology of homicides is characterized by its instrumental nature. The victims are perceived as a means of achieving the aggressor’s main objective, which is to obtain sexual or economic gratification. Likewise, it is possible that there will be no evidence, since the perpetrator may have removed all incriminating evidence, such as starting a fire at the crime scene. Furthermore, these actions are representative of the sexual nature of the crime, where the aggressor may kill the victim due to fear or to avoid subsequently being caught, and also in response to victim resistance. This typology also includes homicides that take place during the course of other criminal activities, mainly robberies, where the death may be caused by an attempt to obstruct the aggressor from obtaining his/her economic reward.

### 3.3. Suitability of Instrumental/Expressive Model

Finally, to assess the suitability of this classification model, each of the analyzed cases were classified in a dominant theme or typology. The majority of the homicides were assigned to the expressive-impulsive profile (86.38%; 387/448 cases), followed by 6.25% of the homicides that had a dominant expressive-family-based theme (6.25%; 28/448 cases); the expressive-distancing and the instrumental-opportunist profile represented 3.13% of the homicides, respectively (14/448 cases per profile), and, finally, only 1.12% of the homicides were assigned to the instrumental-gratification profile (5/448 cases). It should be highlighted that the expressive homicides (expressive-impulsive, expressive-distancing, and expressive-family) explained almost 95% of the simple homicides in Spain between 2010 and 2012.

## 4. Discussion

The results obtained are consistent with prior works that showed that the characteristics of the homicides and the behavior of the aggressors on the crime scene may be differentiated thematically in terms of instrumentality and expressivity, offering an explanation of violent behavior and the interpersonal dynamics between victim and aggressor [30,36,38,39]. Similarly, the effectiveness of the instrumental/expressive model has been shown for establishing a homicide typology that can be linked with the characteristics of the aggressors, using a combination of multivariate statistical techniques [28,29,33,37]. In this way, three subtypes of expressive homicide were identified (expressive-impulsive, expressive-distancing, and expressive-family) along with two subtypes of instrumental homicides (instrumental-opportunist and instrumental-gratification) [26,27,28].

The expressive-impulsive typology is considered to be the most basic form of violent behavior, being the most predominant characteristics of the studied cases [36]. This type of homicide, as found in prior studies, in which the homicide is caused by an argument or conflict with the victim, is carried out without premeditation or planning, due to a lack of impulse control and a strong emotional activation, so, the relevant role of emotions may be explained by the close relationship between the victim and perpetrator [29,33,42]. The profile of the perpetrator that is associated with this type of homicide is male, Spanish, between 31–50 years of age, with a criminal/police record who kills other Spanish men.

Likewise, three subtypes of expressive-impulsive homicides have been identified, associated with different perpetrator profiles. The first refers to non-Spanish national perpetrators aged between 18 and 30 who kill non-Spanish national victims of similar ages. The second refers to offenders having past criminal records for crimes against people, who commit the homicide in exterior locations, flee from the crime scene, carry an armed weapon, and dispose of the same after the crime. The characteristics of the modus operandi coincide with those observed in prior works, finding that due to their increased criminal experience, they are seen to have more forensic knowledge and engage in more precautionary acts [27,28,29,34,37]. The third refers to intimate partner homicides, with the perpetrator being over the age of 51 and the victim tends to be a female of a similar age. The most characteristic aspect of this subtype is that the perpetrator, who, after committing the homicide, tends to commit suicide or attempt to do so [35]. This reactive violence type is due to frustrations that the offender considers to be threats to his own self-esteem, making it likely that they are carried out for revenge or as the result of regular violence [37,47].

The expressive-distancing typology is characterized by the presence of actions that take place after the homicide, which are related to the manipulation of the crime scene and the hiding and displacement of the victim’s body away from the original crime site. It is similar to one of the typologies established by Santtila et al. [36], in which the actions reflect the aggressor’s need to dispose of the crime’s evidence and to remove any connection to the same; in addition, the use of mechanisms to control the victim suggests the possibility that the victim and the aggressor knew each other previously [27]. Therefore, it is possible that homicides identified as expressive-distancing can overlap with instrumental violence in the context of domestic violence, a crime that involves close relationships but is instrumental in that it is driven by power dynamics. Thus, like the “Planned-Expressive theme” suggested by Park et al. [33], this type of homicide can be related to crimes where an angry offender plans his/her offence, such as a victim of intimate partner violence who kill the abuser.

The expressive-family typology refers to homicides carried out within the family environment, in which the perpetrator is a female and the victim is a minor (filicide). Suffocation is the most common homicide method used, suggesting a strong emotional connection between the victim and the perpetrator [21,27]. This typology coincides with that established by Santtila et al. [37], who proposed that it is possible for the aggressor to suffer from some sort of psychological disorder, so the homicides may be fruit of an emotional discharge and it is very likely that the homicide act was caused by frustrations due to personal failures.

The instrumental-opportunist typology includes homicides that are the result of an ulterior motive to the violent act, mainly due to a need for power or control. Victims are strangers or had another sort of relationship with the perpetrator (e.g., prostitutes), therefore, it is possible that the victims, who are especially vulnerable, are perceived as objects by the aggressor, who has no feelings for them [28]. These results coincide with the typologies established in prior works, characterized by highly cognitive behavior, in which the perpetrator acts with a high level of forensic knowledge [29,42].

Finally, in the instrumental-gratification typology, the main motivation of the aggressor is economic or sexual gratification and the instrumental nature of the actions are perceived in the manner by which the perpetrator considers the victim to be a means of satisfying his needs, in line with other prior works [28,36]. According to Sea and Beauregard [42], there is no evidence that the crime was planned in advance, but rather, it is possible that it was caused by the victim’s resistance who, generally speaking, was a stranger. Like the “Instrumental-sex/forensic knowledge” and “Instrumental-robbery” themes identified by Gerard et al. [31], this type of homicide takes place during the course of a sexual aggression or robbery. Similarly, confirming the results of Salfati and Park [33], the perpetrator tends to get rid of incriminating evidence by starting a fire at the crime scene and/or on victim’s body. However, it is possible that homicides identified as instrumental-gratification can overlap with instrumental-opportunist typology, given that gratification can be achieving control.

Our results suggest that all of the examined cases were correctly assigned to the established typologies. Interestingly enough, homicides with an expressive theme were found to explain almost 95% of the cases under study in Spain, with the majority of the homicides taking place between individuals who know one another (friends, family members, couples or intimate ex-partners). In addition, there was an impulsive and emotional nature of these events, such as the result of an interpersonal confrontation with the victim. While this expressive modality is considered to be the most basic form of violent behavior and it would be expected that the majority of the events would be identified with this topic, when comparing our results to those from past studies, we see that in Spain, unlike other countries such as the UK, Finland, Greece, Belgium, Korea, and the US, the expressive theme significantly predominates over the instrumental one, with few cases of homicide having a sexual or economic motivation.

These results, however, have certain limitations. First, the typologies are not clearly defined and can overlap with another. This may be due to the fact that the results were obtained from information from police reports and the database did not include detailed references on the homicide scene, location of injuries, circumstances in which the body was found, and variables related to the victim and the perpetrator were limited, given that this information is not usually included in the police reports since it has no specific purpose for the criminal investigation. Therefore, this information would be necessary in order to enrich the database with information from other sources, such as collecting psychosocial information on the perpetrator through reports created by penitentiary institutions or collecting information on the victims by interviewing individuals from their closest surroundings. So, futures studies should include these variables in order to establish well-defined, more rigorous typologies based on the relationships between the modus operandi, characteristics of the victims, and characteristics of aggressors. Second, the conclusions may not be generalized to all types of homicide, since only simple homicides and those with perpetrators over the age of 18 were considered. Third, there is always the likelihood that human error in the initial data coding may have occurred.

Future research lines may include replications of the methodology used in distinct homicide samples (multiple homicides, juvenile homicides). It may also be interesting to use other theoretical frameworks such as the action system, which simultaneously considers the dynamic processes that connect the individual to the external world (physical and social) and their internal processes (psychological), in order to make inferences on the behavior of the homicides in the crime scenes and to differentiate between criminal acts that connect with the characteristics of the perpetrators [5,61,62].

## 5. Conclusions

In this study, the validity of the instrumental/expressive model has been established in order to thematically differentiate between the structure of simple homicides in Spain, and to establish typologies based on relationships between the modus operandi, the characteristics of the victims, and the characteristics of the perpetrators, thereby contributing to the empirical evidence so as to complement past studies carried out in other cultures and countries, such Belgium [31,32], Korea [33,42], the US [30,34], Finland [36,37], the UK [27,28], and Serbia [29]. Clearly, there is considerable utility in the use of theoretical frameworks that establish a homicide classification offering a psychological explanation of the violent behavior and the dynamics of interaction between victim–offender during the crime. 

This study opted for a research perspective in criminal profiling which, based on empirical evidence, used a large volume of solved cases to generalize conclusions for unresolved cases (inductive criminal profiling). This highlights the value of updating large databases to include all available information regarding how the homicides take place and the characteristics of the crime scene, of the victims, and of the perpetrators in order to establish more detailed and reliable typologies that are of operational utility for unresolved homicide cases.

Similarly, it reveals the importance of examining homicide at a multivariate analysis level, since this is an especially complex crime phenomenon that requires the consideration of collective relationships between variables of interest in order to ensure an increased understanding and to draw useful and applicable conclusions. 

In conclusion, these results have practical implications within the framework of criminal profiling as a complementary tool for assisting police investigations, especially to establish a more rigorous suspect prioritization and to improve human and material resource management, while also helping to reduce the time and economic resources devoted to criminal investigations. The correspondence of several sociodemographic characteristics of the perpetrator and the behaviors carried out at the crime scene across nMDS and cluster analysis may provide some tentative links for prioritizing suspects in Spanish homicide investigations. For example, expressive-family perpetrators were more likely to be a woman and kill minors from the family environment. expressive-impulsive perpetrators were more likely to be Spanish man, between 31–50 years, have a criminal history, and know their victims. Similarly, a subtype of expressive-impulsive homicides is more often perpetrated by offenders over 51 years who kill their partners. Likewise, instrumental-opportunist homicides are more often committed by strangers to the victim. Despite the fact that the Spanish armed forces currently have homicide investigation departments and other groups that specialize in analyzing criminal behavior that are run by police agents with considerable academic and professional experience, this type of study may be used to help train new professionals for these specific investigation departments.

## Figures and Tables

**Table 1:** Variables used in the instrumental/expressive model and their definitions.

Variable Name	Name	Definition
Crime Scene Indoor	INDOOR	Homicides carried out in scenes that are protected from natural elements
Crime Scene Outdoor	OUTDOOR	Homicides carried out in scenes that are exposed to nature
Crime Scene Vehicle	VEHICLE	Homicides carried out inside vehicles
Method of approach	MET_APPR	Method of approaching the victim (includes sudden attack, tricking, prior relationship, or surprise).
Method of control	MET_CONTR	Method used to control the victim (includes aggression, gagging, tying up, use of bodily force of the aggressor, or the presence of a weapon).
* Escaped on foot	ESC_FOOT	The aggressor escapes from the crime scene by foot
* Arrested in crime scene	ARRESTED	The aggressor is detained at the crime scene
* Escaped by vehicle	ESC_VEH	The aggressor uses any type of vehicle to escape from the crime scene
Sharp weapon	SHARP	A weapon that is made up of a metallic blade or another material having similar physical characteristics, for cutting or puncturing.
Firearms	FIREARMS	All portable weapons that have a barrel and that are shot, that are designed to be shot, or that may be easily transformed to shoot a pellet, bullet, or projectile, by action of a combustible propellant
Physical force	FORCE	The aggressor uses his/her physical force to mortally harm the victim
Suffocation	SUFFOCATION	Refers to actions in which an instrument is used to suffocate the victim
Blunt weapon	BLUNT	An object that lacks a sharp edge and/or blade and that may have dull edges that can be used to hit and cause traumatic injuries.
Incendiary weapons	INCENDIARY	Substances, mixtures, and objects in contact with other substances that produce a strong exothermic reaction and may cause fires
Other weapons	OTHER_WEAPON	Any other means used to commit the homicide that is not described in the previous categories
Bring a weapon to crime scene	BRING_WEAPON	Weapon brought to the crime scene by the aggressor to carry out the act
Weapon from crime scene	OPPORTUNITY	Weapon found at the crime scene or in its surroundings and that was not brought by the perpetrator
Located weapon	LOC_WEAPON	The homicide weapon was located by the police agents
* Weapon displacement	WEAPON_DISPL	The weapon was displaced by the aggressor from the crime scene
Body displacement	BODY_DISPL	The body was displaced from the crime scene
Hidden body	HIDDEN_BODY	The victim’s body was hidden by the aggressor
Forensic Awareness	FAWARENESS	Existence of forensic knowledge by the aggressor (specialized knowledge permitting the perpetrator to successfully commit the crime or remove evidence)
Staged	STAGED	Intentional staging of the crime scene by the perpetrator, to mislead the investigation or to make the homicide appear to have been a suicide
Sexual assault	SEXUAL	Sexual assault
Stole	STOLE	Robbery of objects with physical force, robbery with violence and intimidation, and/or robbery and theft of vehicles
Arson	ARSON	Intentional fire set to the crime scene
* Offender aged 18–30 years	OFF_18_30	Perpetrator is between the age of 18–30 years
* Offender aged 31–50 years	OFF_31_50	Perpetrator is between the age of 31–50 years
* Offender aged over 51 years	OFF_+51	Perpetrator is over the age of 51
Male offender	MALE_O	Male offender
Female offender	FEMALE_F	Female offender
* Spanish offender	SPANISH_O	Spanish offender
* Foreign offender	FOREIGN_O	Non-Spanish national offender
Offender’s criminal record	CRIMR	Criminal record and/or police record
Offenders convicted for crimes against the person	CRIMR_PERSON	Record of history of crimes against persons (including homicides)
* Suicide/Attempt	SUICIDE_ASUIC	Suicide carried out or attempted by the aggressor (be it at the crime scene or at another location.)
Minor victim	MINOR_VIC	Victim is under the age of 18
Victim aged 18–30 years	VIC_18_30	Victim is between the ages of 18–30
Victim aged 31–50 years	VIC_31_50	Victim is between the ages of 31–50
Victim aged over 51 years	VIC_+51	Victim is over the age of 51
Male victim	MALE_V	Male victim
Female victim	FEMALE_V	Female victim
* Spanish victim	SPANISH_V	Spanish victim
* Foreign victim	FOREIGN_V	Non-Spanish national victim
Acquaintances	ACQUAINTANCES	Acquaintance/neighbor, friend, work/commercial, school relations
Family	FAMILY	Victim and perpetrator have some sort of family relationship
Stranger	STRANGER	The victim and perpetrator are strangers
Intimate relationship	INTIMATE	Past or present intimate relationship (be it a couple, spouse, ex-couple, separated, or divorced)
Other relationship	OTHER_RELA	Had another type of relationship that is not specified in the previous categories

* indicates that they are variables that have not been used in past studies, but that are included in this study given their special interest for the research.
